# Editorial: Drug repurposing for cancer treatment: current and future directions

**DOI:** 10.3389/fonc.2025.1550672

**Published:** 2025-02-11

**Authors:** Marios Lampros, Nikolaos Vlachos, Georgios D. Lianos, Christina Bali, George A. Alexiou

**Affiliations:** ^1^ Department of Neurosurgery, University of Ioannina, Ioannina, Greece; ^2^ Department of Surgery, University Hospital of Ioannina, Ioannina, Greece

**Keywords:** antipsychotics - treatment, cancer treatment, gastrointestinal cancer, glioblastoma, chemotherapy agents

Cancer is a leading cause of morbidity and death worldwide, with approximately 20 million new cancer cases reported annually (1). Chemotherapy, surgery, and radiotherapy are the mainstay of cancer treatment (2). Despite significant progress in the development of novel chemotherapy agents, the process of developing new chemotherapy agents is time- and money-consuming. In specific, it is estimated that in average, the development of a new drug requires 10 years from discovery to market delivery and approximately 1 billion dollars, while only a minority of the initially promising drugs receive market approval (3). Drug repurposing for cancer treatment is an optimal alternative since promising repurposed drugs have been previously tested for their safety and pharmacokinetic properties and may be directly tested in phase 3 clinical trials (4).

Antipsychotic drugs are psychotropic medications utilized primarily for the treatment of psychotic disorders, including schizophrenia and bipolar disorders (5). These drugs act primarily by mediating the positive psychotic symptoms by inhibiting dopamine receptors in the mesolimbic dopaminergic pathway (5, 6). The first generation of antipsychotic drugs, also called typical antipsychotics, act by inhibiting D2 dopamine receptors (DRD2), while the second-generation antipsychotic drugs act by inhibiting DRD2 and serotonin, 5-HT2A receptors. The observation that the incidence of cancer was reduced in patients receiving chemotherapeutic medications compared to general population led to the assumption that antipsychotic medication may have anti-cancer properties. To date, several studies have been performed to study the potential anti-cancer mechanisms of the antipsychotic medications (6).

## Haloperidol

Haloperidol is the most frequently utilized first-generation antipsychotic medication and today is primarily utilized for the treatment of acute psychosis and delirium (7). It is a non-selective inhibitor of DRD2 and has an affinity for multiple receptors, including anti-muscarinic, anti-adrenergic, and anti-histaminic properties (7, 8). Regarding its role as a repurposing drug, it has been tested for its anti-cancer and anti-microbiological effects (8). Haloperidol has been found to induce apoptosis, mediate autophagy, and cause cell cycle arrest in several cancer types, including glioblastoma and pancreatic cancer (9). Several potential mechanisms have been described, including the reduction in the expression of adhesion molecules in cancer cells, the downregulation of Sonic Hedgehog Pathway (SHH), and the Extracellular signal-regulated kinase pathway (ERK) (6). Moreover, haloperidol has a synergic effect with the alkylating agent temozolamide, the primary chemotherapeutic agent utilized in patients with glioblastoma. In a recent study, Shi et al. reported that this synergic effect was caused by increasing endoplasmic reticulum stress, which leads to autophagy and ferroptosis (10).

## Trifluoperazine

Trifluoperazine is another typical antipsychotic that has been found to have anti-neoplastic properties and suppress tumor growth in several types of cancer, including glioblastoma, hepatocellular carcinoma, breast, colorectal, and lung cancer (6). Regarding its effect on glioblastoma, trifluoperazine has been found to enhance the radiosensitivity of glioma cells *in vitro* via decreasing the radiation-induced-Nanog expression (11). Moreover, trifluoperazine was found to induce the activation of FOXO1 transcription factor in lung cancer, hepatocellular carcinoma, and glioma cell lines that suppress the expression of multi-drug resistance (MDR) genes, including P glycoprotein, and to increase the sensitivity of these cancer cells in chemotherapy agents (12–14).

## Chlorpromazine

Chlorpromazine is a phenothiazine class antipsychotic drug associated with anti-cancer effects in glioblastoma, white blood cell malignancies, oral cancer, Ewing sarcoma, and colorectal cancer (6, 15–17). Specifically, chlorpromazine has been found to decrease cell growth and induce autophagic cell death and apoptosis (15, 16) In glioma cell lines, chlorpromazine was found to downregulate the expression of stemness genes such as NANOG and promote autophagy via inhibition of the Akt/mTOR pathway (16, 17). Additionally, chlorpromazine induces apoptosis in various leukemia types, including B and T cell malignancies, by inhibiting the mitochondrial DNA polymerase (15). Finally, chlorpromazine induced a mitotic arrest via inhibition of mitotic kinesin *in-vivo* and *in-vitro* in colorectal cell lines (18).

## Penfluridol

The repurposing role of penfluridol as an anti-cancer medication has been experimentally studied in multiple studies in various cancer types, including melanoma, glioblastoma, breast, pancreatic, and lung cancer (6, 19). Mechanistically, penfluridol induces apoptosis and autophagy and inhibits cancer cell invasion. Interestingly, penfluridol has been found to downregulate integrin expression, thus reducing cancer cells’ invasiveness and metastatic potential (20, 21). Moreover, penfluridol has been found to reduce the expression of GLI-1, a transcription factor overexpressed in glioma cells involved in chromatin regulation. GLI-1 downregulates the expression of transcription factors such as SOX2 and reduces the expression of proteins involved in cell invasion, including integrins and epithelial to mesenchymal transition (22). Penfluridol also suppresses the HER2/b-catenin signaling pathway in breast cancer cell lines. HER2/-catenin is an important pathway involved in cell survival, and this pathway is the pharmaceutic target of paclitaxel, a widely utilized chemotherapeutic drug. Of note, Gupta et al. found that penfluridol enhanced the efficacy of paclitaxel in *in-vivo* breast cancer models (23).

## Thioridazine

Thioridazine is a phenothiazine antipsychotic drug that is probably the most studied antipsychotic drug regarding its role in multiple cancer types, including breast, lung, gastrointestinal, hepatocellular, prostate, renal, breast, testicular, hematological, and gynecological cancers (6, 24–26). The anti-tumor effect of thioridazine seems to be mediated through the suppression of angiogenesis, cell cycle arrest, induction of autophagy, and apoptosis (6, 24). Suppression of angiogenesis is mediated by the inhibition of Vascular Endothelial Growth Factor (VEGF) via various mechanisms, including downregulation of PI3/Akt and VEGF/PIEK/mTOR pathway and inhibition of Hypoxia Inducing Factor (HIF)-1a (24, 27, 28). Thioridazine-induced downregulation of PI3/Akt pathway in cancer cell lines has also induced autophagy, apoptosis, and cell cycle arrest. The promising results of experimental studies led to the conduction of a Phase 1 clinical trial evaluating the effect of the combination of thioridazine and cytarabine in acute myeloid leukemia, in which a reduction of about 50% in the blast cells was found in most patients (29).

## Typical antipsychotics

Several other typical and atypical antipsychotics have been studied regarding their potential role in cancer treatment. To date, the main body of research is currently focused on typical antipsychotics compared to atypical antipsychotics. The latter is probably explained by the increased affinity of typical antipsychotics for DRD2 receptors, which are implicated in tumorigenesis. However, during the last decade, increased reports regarding the potential role of atypical antipsychotics in cancer treatment have been accumulated. Olanzapine is a first-line atypical antipsychotic agent widely utilized for various psychiatric diseases worldwide. Olanzapine has been reported to enhance the therapeutic effect of temozolomide in glioblastoma cell lines (30). A similar synergic effect of temozolomide with quetiapine in glioma cell lines, another first-line antipsychotic drug, has also been observed (31). Preliminary promising results were also reported for various cancer types for other atypical antipsychotics, including risperidone for prostate (32) and breast cancer (33), aripiprazole for breast (34), and lung cancer (35). Finally, clozapine, a very effective antipsychotic medication for resistant psychosis but with an unfavorable safety profile because it is associated with significant adverse effects, including neutropenia and cardiomyopathy, has been found to induce G0/G1 cell cycle arrest in lung cancer (36) and melanoma cell lines (37).

## Synergic effect of antipsychotics with chemotherapy drugs

Except for their experimental study as sole anti-cancer medication, antipsychotics are frequently studied for their synergic effect with chemotherapy drugs (6) Temozolomide (TMZ) is an alkylating agent broadly utilized as an adjuvant glioblastoma treatment (6, 10). Haloperidol, penfliridol, quetiapine, and olanzapine are among the antipsychotic drugs that have been found to have a synergic effect with TMZ in the treatment of GBM (10, 38). Several mechanisms have been proposed to explain this effect, including inhibition of the extracellular signal-related kinase (ERK), Cco subunit 4 isoform 1 (COX4-1), and Cx43, a molecule essential in DNA repair (22, 31, 38). Doxorubicin is an anthracycline-based chemotherapy medication widely used to treat hematologic and solid cancers. The cytotoxic effect of doxorubicin seems to be enhanced when combined with trifluoperazine via a mechanism that involves transnuclear translocation of FOXO1, a transcription factor that suppresses the expression of multidrug resistance (MDR) genes (13, 39).

## Repurposing antipsychotics and toxicity

Despite the efficacy of antipsychotic medication in the treatment of various psychiatric disorders, their use is associated with significant side effects (40). First-generation antipsychotics, or typical antipsychotics, cause significant sedation and extrapyramidal symptoms mediated via inhibition of the D2 receptors. Thus, their broad use in the past caused impairment in the quality of life of patients with psychiatric disorders (41). The development of the second generation, or atypical antipsychotics, which have a more favorable side effect profile with minimal extrapyramidal symptoms, led to the replacement of the typical antipsychotics (42). However, atypical antipsychotics also cause some degree of sedation and are associated with metabolic disturbances, including weight gain and risk for insulin resistance and diabetes development (6, 42). These factors, in addition to the antipsychotic-related stigma in society, may be potential factors that could limit the enrollment of cancer patients in future promising clinical trials (43). Future studies should consider all the limitation and toxicity associated with the use of antipsychotics as anti-cancer medication. However, the pharmacokinetic properties and the toxicity of antipsychotic drugs are well recognized by both clinicians and researchers due to their broad use (44). Hence, optimal dose adjustment and symptoms management may not be a significant obstacle. Moreover, recent data support that the use of typical antipsychotics may improve the anorexia of patients with advanced cancer and may be utilized as a part of palliative treatment (45).

## Current state and future perspectives

Over the last two decades, mounting evidence from experimental studies has indicated the promising role of antipsychotics in cancer treatment. Antipsychotics seem to have a dual role in cancer treatment: They directly inhibit tumor growth, induce apoptosis, and enhance the sensitivity of chemotherapy agents. However, despite the increased pre-clinical evidence, only three early-phase (I/II) clinical trials (NCT04224441, NCT02096289) have been conducted so far. One potential reason for the latter is that typical antipsychotics, the leading group of antipsychotics targeted for cancer repurposing, have significant side effects such as extrapyramidal symptoms, sedation, and anticholinergic effects, that may not be optimal for cancer patients with an increased morbidity. Hence, more pre-clinical and clinical studies should be performed both in first and second-generation antipsychotics to clarify the exact role of antipsychotics as repurposing drugs for cancer treatment.
